# Development of a rapid point-of-care patient reported outcome measure for cataract surgery in India

**DOI:** 10.1186/s12955-018-0855-5

**Published:** 2018-01-30

**Authors:** Joshua R. Ehrlich, Charlie Frank, Josiah Smiley, Hong-Gam Le, Sanil Joseph, Stephen G. Schilling, Brian C. Stagg, Joshua D. Stein, R. D. Ravindran, Aravind Haripriya

**Affiliations:** 1 Department of Ophthalmology and Visual Sciences, University of Michigan, 1000 Wall Street, Ann Arbor, MI 48103 USA; 2 Institute for Healthcare Policy and Innovation, University of Michigan, Ann Arbor, MI USA; 3 Center for Eye Policy and Innovation, University of Michigan, Ann Arbor, MI USA; 4 Lions Aravind Institute of Community Ophthalmology, Aravind Eye Care System, Madurai, Tamil Nadu India; 5 Institute for Social Research, University of Michigan, Ann Arbor, MI USA; 6 Department of Physical Medicine and Rehabilitation, University of Michigan, Ann Arbor, MI USA; 7 Department of Health Management and Policy, University of Michigan, Ann Arbor, MI USA; 8 Aravind Eye Care System, Madurai, Tamil Nadu India

**Keywords:** Ophthalmology, Cataract, Rasch analysis, Patient-reported outcome measures

## Abstract

**Background:**

For patient undergoing cataract surgery in India, existing patient-reported outcome (PRO) measures are either not culturally relevant, have not been adequately validated, or are too long to be used in a busy clinical setting. We sought to develop and validate a brief and culturally relevant point-of-care PRO measure to address this need.

**Methods:**

Twelve items from the Indian Visual Functioning Questionnaire (IND-VFQ) were selected based on preliminary data. Patients 18 years and older were prospectively recruited at Aravind Eye Care System in Madurai, India. Clinical and sociodemographic data were collected and the 12-item short-form IND-VFQ (SF-IND-VFQ) was administered pre- and post-operatively to 225 patients; Factor analysis and Rasch modeling was performed to assess its psychometric properties.

**Results:**

One item that did not fit a unidimensional scale and had poor fit with the Rasch model was eliminated from the questionnaire. The remaining 11 items represented a single construct (no residual correlations> 0.1) and were largely unaffected by differential item functioning. Five items had disordered thresholds resolved by collapsing the response scale from four to three categories. The survey had adequate reliability (0.80) and good construct (infit range, 0.77–1.29; outfit range, 0.56–1.30) and content (item separation index, 5.87 logits) validity. Measurement precision was fair (person separation index, 1.97). There was evidence that items were not optimally targeted to patients’ visual ability (preoperatively, − 1.92 logits; overall, − 3.41 logits), though the survey measured a very large effect (Cohen’s *d* 1.80). In a subset of patients, the average time to complete the questionnaire was 2 min 6.3 s.

**Conclusions:**

The SF-IND-VFQ is a valid, reliable, sensitive, and rapidly administered point-of-care PRO measure to assess changes in visual functioning in patients undergoing cataract surgery in India.

**Electronic supplementary material:**

The online version of this article (10.1186/s12955-018-0855-5) contains supplementary material, which is available to authorized users.

## Background

In the United States [1] India [2] and elsewhere there is a growing emphasis on understanding the impact of medical interventions on the lives of patients to assess and improve quality of care. In the case of cataract surgery, success is often equated with an improvement in an individual’s best-corrected visual acuity (BCVA). However, it is also meaningful to judge the success of surgery in relation to vision-related quality of life (QOL) and vision-dependent functioning, as these factors likely drive patients to pursue surgery and affect their satisfaction.

Many patient-reported outcome (PRO) measures have been used to assess the impact of cataract on vision-related QOL and functioning [3–9] and a number of these have been employed among Indian patients undergoing cataract surgery [8–11]. However, existing instruments may not be culturally relevant to patients in India [3, 4]; have not been adequately validated [9]; or are too lengthy to be deployed as point-of-care tests in a high-volume clinical setting [7].

Among existing PRO measures, the Indian Visual Functioning Questionnaire (IND-VFQ) was developed in India using rigorous qualitative methodology [12]; was validated with Rasch analysis [13]; and has been shown to detect a change in vision-related QOL after cataract surgery [10]. Nonetheless, with 28 items, the Rasch-modified version of the IND-VFQ was too long for use at the point-of-care in a busy clinical practice at Aravind Eye Care System (AECS) in Madurai, India. Therefore, we sought to develop a rapidly administered short-form version of the IND-VFQ (SF-IND-VFQ) and to test its psychometric properties for patients undergoing cataract surgery at a tertiary eye hospital in south India. Our purpose in validating a rapid point-of-care PRO for cataract surgery is to facilitate patient-centered measurement for quality improvement, monitoring of surgical results, and use in future research studies.

## Methods

This study was approved by the AECS Institutional Review Board and adhered to all tenets of the Declaration of Helsinki.

### Item selection

The original IND-VFQ was developed around the qualitative insights of patients who participated in 46 focus groups across three different regions of India, including Tamil Nadu [12]. Investigators then generated and tested 45 survey items among subjects with cataract, glaucoma, diabetic retinopathy and age-related macular degeneration [7]. Based on an evaluation using classical test theory, they produced a final 33-item version of the IND-VFQ with four subscales (mobility, activity limitation, psychosocial impact, and visual symptoms). Subsequently, the instrument was refined to a 28-item version based on the results of a Rasch analysis in which the IND-VFQ was administered to patients in Tamil Nadu with cataract [13].

We used the 28-item IND-VFQ [13] as the basis for the development of the SF-IND-VFQ. In an exploratory first phase, we administered the 28-item survey to an independent sample of 223 patients at AECS in Madurai, Tamil Nadu, India prior to and one month after cataract surgery. For each item, we calculated the Cohen’s *d* effect size (*d* = [mean_1_ – mean_2_ / SD]) [14]. We considered for inclusion in our pilot instrument any item with *d* ≥ 0.5, which is considered a moderate effect size [14], and for which a minimum of 40% of subjects indicated that they were at least minimally affected before surgery (any response other than “not at all”). Eleven items met these criteria. An expert panel of ophthalmologists (AH, JDS, JRE) then selected one additional item from the activity limitation domain to ensure that each domain was represented by at least 2 items. This resulted in the 12 item pilot questionnaire that we evaluated in this study.

### Subjects

We recruited patients in the free and paid wards at AECS Madurai from June–July 2016. All patients had previously undergone a full ophthalmologic examination at the AECS Cataract and Intraocular Lens Service and had chosen to pursue cataract surgery. Criteria for inclusion were: ≥ 18 years of age; undergoing routine cataract surgery (by phacoemulsification or manual small incision cataract surgery [MSICS]); and planning to return to AECS Madurai for a one-month post-operative examination. Subjects were excluded if they: had impairment that precluded communication with an interviewer; did not speak Tamil; or lived > 50 km from Madurai since these patients commonly seek post-operative care at Aravind satellite clinics. No patients who completed the parent 28-item IND-VFQ in the exploratory phase contributed data to the validation phase of the study.

To recruit patients, the surgery roster for the day was reviewed each morning and those meeting study criteria were noted. Patients were approached in the triage area, told about the study, and informed consent was obtained. A research assistant was present to assist subjects in completing the survey if needed. An observer timed a subset of consecutive patients while they completed the survey either with or without assistance. To collect postoperative data with minimal loss to follow-up, we tagged medical charts of study patients and the study team received an alert when patients returned for their post-operative visit. Responses were reverse coded so that lower values represented poorer visual functioning. We sought a minimum sample size of 150 based on empirical evidence that this would provide person measures stable within ± ½ logit with 99% confidence [15].

### Demographics and clinical measures

We recorded best-corrected visual acuity (BCVA) at each exam using a Snellen chart at 6 m with standard overhead illumination; testing distance was varied if vision was too poor to test at 6 m. Visual acuities were converted to log minimum angle of resolution (logMAR) values. LogMAR values for counting fingers and hand motions were recorded as 2.0 and 3.0, respectively [16]; perception of light was also recorded as logMAR 3.0 since no standard value existed for this level of vision. The following sociodemographics were collected: age, sex, place of residence, marital status, education, employment status, monthly household income, and whether surgery was paid for or free.

### Analyses

Rash analysis, a form of modern test theory, has emerged as the gold standard methodology for validating visual-functioning questionnaires [17]. In contrast to classical test theory, Rasch analysis accounts for both the difficulty of tasks and the abilities of subjects by modeling the relationship between a latent trait (i.e. a patient’s functional ability) and the items used to measure that trait [18]. Therefore, a Rasch analysis produces estimates of item difficulties, which correspond to the amount of visual ability required by a given task, and person abilities, which are a measure of participants’ ability to perform tasks that require vision. These can be utilized to estimate interval scales rather than summing Likert scores that assume an equal difference between any two consecutive response categories. The results of a Rasch analysis are then compared to normative values to assess the reliability, validity and measurement precision of a PRO measure [18–21]. Massof has published an extensive discussion of Rasch analysis and its application to PRO measures of visual ability [17].

The Rasch model is based on the assumption of unidimensionality, which describes the measurement of a single latent construct. Therefore, we performed exploratory factor analysis (EFA) by weighted least squares of the polychoric item-test correlation of pre- and post-test scores [22]. We then performed confirmatory factor analysis (CFA) for a one factor model and calculated residual correlations for each item and for model fit indices, including the root mean square error of approximation (RMSEA), comparative fit index (CFI) and Tucker-Lewis index (TLI). Good fit with a one-factor model is indicated by an RMSEA value < 0.08 and CFI and TLI values ≥0.95 [22]. Factor analyses were performed using MPlus (version 7.0).

Next, after performing EFA and CFA, we fit SF-IND-VFQ responses to the Rasch model using Winsteps software (version 3.92.1). The likelihood-ratio test was significant, suggesting the partial credit model (PCM) was applicable to our data since it allows for response thresholds to vary from item to item [23]. We fit our survey response data to the Rasch model to estimate item and overall model fit by comparing our results to normative values that indicate instrument reliability, precision and validity [18–21]. Polyserial item-test correlations were calculated, representing the inferred latent correlation between a continuous variable, the test score, and an ordered categorical variable, the item score. For each survey item, we calculated estimates of visual functioning for each response category; response category thresholds; and fit statistics. For all analyses, data from the pre- and post-operative periods were pooled.

In order for data to fit the Rasch model well, there should not be differential item functioning (DIF) [17]. This occurs when item responses vary among subgroups of the population; for example, when men and women have similar visual ability but respond differently to the same item. We performed testing for DIF using logistic regression and the magnitude of DIF was measured by McFadden’s pseudo-R^2^ criterion. We examined the following variables for DIF: age, sex, location of home (urban versus suburban/rural), marital status, educational attainment, employment status, household income, pay status (whether surgery was paid for or free), and surgery type (phacoemulsification or MSICS). Finally, to test the responsiveness of our instrument to cataract surgery, we calculated the average change in survey score and Cohen’s *d*, a measure of effect size [14].

## Results

### Subjects

Patient characteristics are presented in Table 1. 225 patients were included with an average age of 60.4 ± 8.6 years and 66.2% were female. About half of patients paid for surgery (51.1%) and underwent MSICS (53.8%), while a majority lived in an urban setting (76.0%); did not complete high school (58.2%); were unemployed (54.2%); were married (71.1%); and had a monthly household income ≤5000 Rupees (about US$78). The mean pre-operative BCVA in the eye undergoing surgery was logMAR 0.46 ± 0.44 (approximately 6/18), which improved to 0.07 ± 0.13 (approximately 6/6) after surgery. The average time to complete the survey for a subset of 18 consecutive patients was 2 min 6.3 s (range, 1 min 9 s to 4 min 37 s).

**Table 1:** Patient Sociodemographics

Subjects	225
Age, mean (SD)	60.4 (8.6)
Female sex	66.2%
Pay status	
Paying	51.1%
Free	48.9%
Surgery Type, %	
Phacoemulsification	46.2%
MSICS	53.8%
Place of living	
Urban	76%
Suburban/rural	24%
Education	
No schooling	36.4%
Primary	21.8%
High school	30.7%
Undergraduate	6.2%
Post-graduate	4.9%
Employment	
Employed	36.0%
Retired	9.8%
Unemployed	54.2%
Marital Status	
Married	71.1%
Widowed	26.2%
Never married	2.7%
Monthly Income, INR ($USD)	
≤ 5000 ($78)	52.0%
5001–10,000 ($156)	20.4%
10,001–20,000 ($311)	8.9%
20,001–30,000 ($467)	3.1%
≥ 30,001	4.4%
No response	11.1%

*INR* Indian Rupees

*USD* United States Dollars

*MSICS* manual small incision cataract surgery

### Dimensionality

EFA showed that the first eigenvalue was the only one that was substantially greater than one, which suggested that the instrument was dominated by a single factor; this is illustrated in the scree plot in Additional file 1. In order to test this, we performed CFA for the one factor model. Item 11, “Does bright light hurt your eyes?” had three residual correlations > 0.1 with other items, suggesting that this item may not fit with the single underlying factor. With this item removed, the RMSEA was 0.077, CFI was 0.98 and TLI was 0.98. Of note, the RMSEA, CFI and TLI statistics with item 11 included were 0.073, 0.98, and 0.98, respectively, indicating similar model fit with and without item 11. There were no additional items with residual correlations > 0.1.

### Rasch analysis

Survey items are listed in Table 2. We examined response frequencies and ordering of response thresholds for each item. Items 1, 2, 3, 5 and 7 all had evidence of disordered thresholds. This meant that for these items successive response categories did not correspond to increasing values of visual ability. Therefore, we collapsed the response scale for these items from four to three categories, after which all items had ordered thresholds. We then fit a PCM model to the recoded items.

**Table 2:** Item Parameter Estimates and Fit Statistics

Item	Item location	Infit MNSQ	Outfit MNSQ	Polyserial item-test correlation
1. Problem climbing stairs	−0.49	0.90	0.63	0.59
2. Problem making out bumps	0.14	0.87	0.76	0.69
3. Problem seeing animals or vehicles	−0.15	0.77	0.56	0.66
4. Problem recognizing faces	−0.27	0.97	0.69	0.58
5. Problem seeing outside in bright light	1.20	1.00	0.94	0.75
6. Frightened to go out	−1.00	0.99	1.06	0.59
7. Enjoy social functions less	−0.37	0.94	0.81	0.55
8. Ashamed can’t see	−0.75	1.06	1.00	0.60
9. Dazzled in bright light	1.38	1.29	1.30	0.70
10. Vision blurred in sunlight	−0.18	0.76	0.69	0.77
11. Bright light hurt eyes^a^	0.03	1.53	2.32	0.48
12. Blurred vision	0.45	0.87	0.87	0.82

*MNSQ* mean-square

^a^item removed to form 11-item SF-IND-VFQ

We examined data for the presence of DIF. We did not detect DIF related to age, sex, location of home, marital status, educational attainment, employment status or household income for any item. Item 2 showed DIF for surgery type, with patients undergoing MSICS being less likely to endorse higher response categories. Item 8 showed non-uniform DIF for pay status, with individuals who had reduced payment or free surgery showing less discrimination than individuals who fully paid for surgery. However, for both items the magnitude of the DIF as measured by McFadden’s pseudo R^2^ criterion was negligible (R^2^ < 0.02).

Table 2 provides a summary of the difficulty and fit of each item. The infit and outfit mean-square (MNSQ) statistics provide evidence of construct validity when expected values are close to 1.0, with values from 0.5 to 1.5 being useful for measurement [20]. Values considerably > 1.0 denote randomness in the data (e.g. noise and outliers), while values < 1.0 imply that an item may not be productive for measurement because its values are too predictable. Item 11 was the only item that exhibited significant misfit, with an infit MNSQ of 1.53 and outfit MNSQ of 2.32.

The item-test correlation for item 11 was also low (0.48) compared to other items. These findings suggest that Item 11, which also did not fit the single factor model, could be removed without affecting measurement precision.

The average visual functioning estimates for each response category of each item are reported in Additional file 2 along with category thresholds and misfit statistics for each of the 12 items. The misfit statistics for each item category correspond to the overall data reported in Table 2. However, for item 6 in which there were observed responses for only two of the categories, we detected category misfit due to the small number of observed values.

Table 3 presents reliability values and separation indices, calculated after having omitted Item 11. We found a high item reliability (0.97), indicating that our sample was large enough to achieve statistically reproducible item difficulties. Person reliability was acceptable (0.80), confirming the overall reliability of the questionnaire. Measurement precision, indicated by the person separation index (1.97 logits), approached the commonly accepted threshold of 2.0 logits and a high item separation index (5.87 logits) specified good content validity.

**Table 3:** Overall Model Fit

Combined pre- and post-operative data					Pre-operative only	
	Separation	Reliability	Mean	SD	Mean	SD
Person Ability	1.97	0.80	3.41	2.23	1.92	1.91
Item Difficulty	5.87	0.97	0.00^a^	0.77	0.00	0.78

*SD* standard deviation

^a^the mean item difficulty was set at 0.00 and the data were fit around this fixed value

**Fig. 1:**
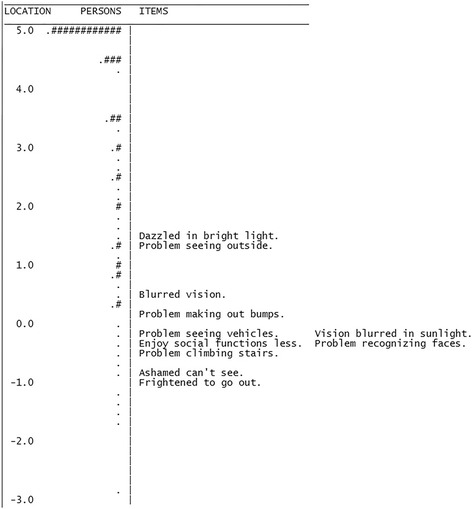
Person-Item Map. The person-item map comparing patients’ visual ability and the visual ability required by each item suggests suboptimal targeting. The mean item difficulty is fixed at 0.00 and the data were fit around this value

**Fig. 2:**
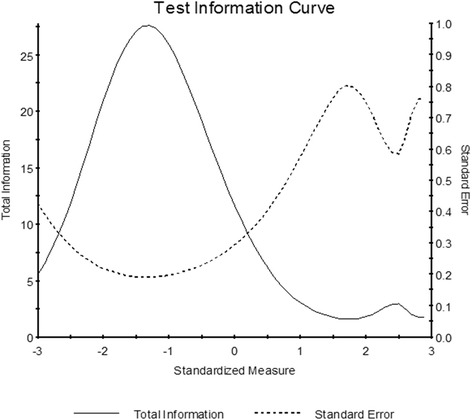
Test-Information Curve. The test-information curve shows that survey scores provide high information and good precision for all values less than zero (the standardized mean of the person distribution) but that information and precision decreases for higher levels of visual ability

We also calculated the mean item difficulty and person ability, which were 0.00 ± 0.77 and 3.41 ± 2.23 logits, respectively. If items are perfectly targeted to a sample, there should be minimal or no difference between person ability and item difficulty. We observed suboptimal targeting, as the visual ability of subjects was greater than that required by the survey items, which were too easy for this population. The person-item map in Fig. 1 illustrates this, showing a mismatch between item and person scores. Furthermore, the test information plot in Fig. 2 demonstrates that items provided the greatest information for the lowest functioning individuals. This plot shows that the SF-IND-VFQ achieved high information and good precision for all values less than zero (the mean of the person distribution), but that information degraded for higher values. Of note, when only pre-operative data were analyzed targeting remained suboptimal, though the mean difference between person and item measures improved considerably to 1.92 logits.

### Effect size

The average change in SF-IND-VFQ scores after cataract surgery was 2.98 ± 0.11 logits. Cohen’s *d* was 1.80, indicating a very large effect size [14].

## Discussion

We found that the 11-item SF-IND-VFQ is a unidimensional instrument capable of detecting changes in visual functioning among Tamil-speaking patients undergoing cataract surgery in India. The culturally-relevant questionnaire was administered rapidly at the point-of-care and had good reliability, content validity and was largely unaffected by DIF.

The purpose of this study was to design a short-form PRO measure that is appropriate as a point-of-care instrument for use in high volume clinical settings in order to monitor outcomes, promote quality improvement, and facilitate outcome measurement in future pragmatic clinical trials and clinical research studies. Traditionally, PRO measures have been employed as research tools and are often too time-consuming to be deployed in a busy clinical setting. However, in more recent years, with the growth of the National Institutes of Health’s Patient-Reported Outcomes Measurement Information System (PROMIS) and the National Health System’s quality improvement initiatives in the United Kingdom, the use of PRO measures in clinical practice has become more common [24].

The SF-IND-VFQ meets most criteria that Kroenke and colleagues proposed for using PRO measures in clinical practice [25]. Briefly, the survey is setting-appropriate based on its cultural relevance and low respondent burden; can be self-administered and quickly completed; has items that focus on a single symptom; evaluates only one dimension (symptom severity), with no more than four response categories per item; and is widely accessible at no cost. Additional criteria such as actionability (translating scores into concrete actions) and universality (usefulness in other conditions) will be tested in future studies as the SF-IND-VFQ is used more broadly throughout AECS.

One item present in the initial SF-IND-VFQ, “Does bright light hurt your eyes?,” was removed since it did not fit the same unidimensional construct as other items and had poor fit with the Rasch model, as denoted by its MNSQ values. Notably, this was the only item that assessed pain symptoms and this may explain why it did not form part of a unidimensional scale. All other items had goodness-of-fit statistics within the acceptable range, which confirmed the instrument’s construct validity.

Two items retained in the SF-IND-VFQ were affected by DIF, though the magnitude of DIF was negligible for both of these. The first of these items (item 2) asked, “because of your vision how much trouble do you have making out the bumps and holes in the road when walking?” Patients undergoing MSICS, but not phacoemulsification, were less likely to endorse response categories associated with high visual functioning. This may be the case since MSICS is frequently the preferred surgical technique for very advanced cataracts that tend to cause more severe visual dysfunction. Item 8, “because of your eye problem are you ashamed that you can't see?” had non-uniform DIF in which patients who received free surgery showed less discrimination than those who paid for surgery. The reason for this finding is less clear but may be related to understanding or interpretation of the question.

The SF-IND-VFQ was able to discriminate effectively amongst individuals with high levels of visual dysfunction. However, overall the instrument demonstrated suboptimal targeting. The mean difference between person ability and item difficulty was 3.41 logits, suggesting that the amount of visual functioning required by SF-IND-VFQ items was not matched to the visual ability of our subjects. Other cataract surgery PRO measures have also suffered from poor targeting because of items being too easy for patients with cataract [26, 27]. One exception is Catquest-9SF, which showed excellent targeting in a Swedish population (preoperatively, − 0.34 logits) [4]. In our current study, since patients had poorer vision before surgery, SF-IND-VFQ targeting was considerably better preoperatively, though it remained suboptimal (− 1.92 logits).

One reason for suboptimal targeting in this study was that the long-form IND-VFQ was also not well-targeted to patients with cataract [13]. The IND-VFQ was developed through focus groups of patients with cataract, glaucoma, diabetic retinopathy, macular degeneration, and “mixed low vision.” Among the 308 participants from three regions of India, about half (51.9%) had cataracts. In a subsequent study, Finger and colleagues surveyed patients with cataract in south India with the initial 33-item IND-VFQ [13]. Using principal components analysis, they found that the survey contained five subscales (general functioning, mobility, activity limitation, psychosocial impact, and visual symptoms). When Rasch analysis was performed, the visual symptoms subscale had the best targeting (− 0.93 logits), while general functioning (− 2.32), mobility (− 2.94), activity limitation (− 1.93) and psychosocial impact (− 1.39) were not as well targeted. We had similar results when we assessed preoperative targeting (− 1.92 logits), which is comparable to the cross-sectional analysis in Finger et al.’s study of patients with cataract [13]. Since subjects in our study had relatively preserved preoperative visual function (mean BVCA of about 6/18) the instrument may be better targeted to cataract patient populations in India with more advanced disease and this could be explored in future studies.

Given the strong psychometric properties of Catquest-9SF, we had considered employing this instrument for our study rather than validating a new short-form questionnaire. However, although Catquest was well-targeted to patients in Sweden [4] and several other settings, targeting had not been optimal in all populations [27]. Additionally, we did not believe that all Catquest items were culturally relevant to our patients at AECS. Specifically, items related to reading newspaper text, doing handicrafts, reading text on television, and carrying out a preferred hobby were not thought to be pertinent.

The SF-IND-VFQ was extremely sensitive to changes in visual function after cataract surgery, with the average score improving by 2.98 logits. Additionally, the effect size in this study, measured by Cohen’s *d*, was 1.80, which is considered *very large* based on accepted criteria [14]. This was due in part to including only IND-VFQ items that were known to be sensitive to the impact of cataract surgery. The effect size for the SF-IND-VFQ was also high in comparison to other cataract PRO measures. McAlinden and colleagues conducted a study in which 16 PROs were administered to patients having cataract surgery in Australia [28]. Out of this battery, they found that Catquest-9SF had the largest effect size with a Cohen’s *d* of 1.45.

### Limitations

Although our findings indicate that the SF-IND-VFQ is a valid, reliable and quickly administered PRO, there were several limitations to our study. First, our findings may not be generalizable beyond a tertiary eye center in south India; however, future work should be done to test the measurement properties of the SF-IND-VFQ in different geographic and eye care delivery settings, such as in a lower resource field-based setting in India. Second, we intentionally selected items from the parent IND-VFQ that were most sensitive to the effect of cataract surgery, though we acknowledge that targeting might have been better had we chosen items using different criteria.

All analyses used combined pre-operative and post-operative data. We acknowledge that this violates the Rasch model’s assumption of independence. However, in pooling data we are able to provide item calibrations that can be applied to patients both before and after undergoing cataract surgery, a feature that makes this approach clinically useful. To determine the impact of this violation of the assumption of independence, we analyzed pretest data alone and compared this to results from our pooled analyses. We found no differences in the results for the EFA or CFA, while Rasch person reliability was 0.78 for pretest data alone compared to 0.80 for the pooled data and the 11 retained items continued to show good fit with the model. Of note, we were unable to fit a separate model for the post-operative data since there were few responses indicating severe visual dysfunction, as one would expect due to patients’ improved vision after undergoing surgery.

## Conclusions

Building on prior work in India [9–11], the current investigation describes the development of a brief and culturally relevant PRO measure that can be quickly administered at the point-of-care. Though further work could be done to improve the targeting of the SF-IND-VFQ, these features suggest that it may be a feasible tool to assess patient-reported visual function in a busy clinical practice. As the original IND-VFQ is available in multiple Indian languages, our short-form version can be easily tested in other parts of India. This questionnaire may prove useful in future work to monitor and improve surgical outcomes and to facilitate clinical research and pragmatic clinical trials at AECS and elsewhere. We have developed a raw-score to Rasch person measure conversion to allow others to use the SF-IND-VFQ without performing their own Rasch analysis (Additional file 3).

## Additional files

Additional file 1: Scree Plot of Exploratory Factor Analysis. This plot illustrates that the first eigenvalue of the exploratory factor analysis was the only value that was greater than 1. This suggests a unidimensional model. (TIFF 929 kb)

Additional file 2: Supplementary tables from Rasch Analysis. These tables report for each of the 12 survey items: the average visual functioning estimates for each response category; category thresholds; and misfit statistics. (DOCX 19 kb)

Additional file 3: Raw score to Rasch person-measure conversion. This worksheet allows researchers and clinicians to covert raw SF-IND-VFQ scores to interval scores without performing their own Rasch analysis. Caution should be exercised in applying this Rasch analysis to patient populations that are very different from the one in this study. (XLSX 12 kb)

## Abbreviations

AECS: Aravind Eye Care System
BCVA: Best-corrected visual acuity
CFA: Confirmatory factor analysis
CFI: Comparative fit index
DIF: Differential item functioning
EFA: Exploratory factor analysis
IND-VFQ: Indian Visual Functioning Questionnaire
INR: Indian Rupees
logMAR: Log minimum angle of resolution
MNSQ: Mean-square statistic
MSICS: Manual small incision cataract surgery
PCM: Partial-credit model
PRO: Patient-reported outcome
PROMIS: Patient-Reported Outcomes Measurement Information System
RMSEA: Root mean square error of approximation
SD: Standard deviation
SF-IND-VFQ: Short-Form Indian Visual Functioning Questionnaire
TLI: Tucker-Lewis index
USD: United States Dollars

## References

[1] Basch E (2017). Patient-reported outcomes — harnessing patients’ voices to improve clinical care. N Engl J Med.

[2] Dang A, Mendon S (2015). The role of patient reported outcomes (PROs) in healthcare policy making. Syst Rev Pharm.

[3] Steinberg EP, Tielsch JM, Schein OD, Javitt JC, Sharkey P, Cassard SD (1994). The VF-14. An index of functional impairment in patients with cataract. Arch Ophthalmol.

[4] Lundstrom M, Behndig A, Kugelberg M, Montan P, Stenevi U, Pesudovs K (2011). The outcome of cataract surgery measured with the Catquest-9SF. Acta Ophthalmol.

[5] Pesudovs K, Gothwal VK, Wright T, Lamoureux EL (2010). Remediating serious flaws in the National eye Institute visual function questionnaire. J Cataract Refract Surg.

[6] Mangione CM, Orav EJ, Lawrence MG, Phillips RS, Seddon JM, Goldman L (1995). Prediction of visual function after cataract surgery. A prospectively validated model. Arch Ophthalmol Chic Ill 1960.

[7] Gupta SK, Viswanath K, Thulasiraj RD, Murthy GVS, Lamping DL, Smith SC (2005). The development of the Indian vision function questionnaire: field testing and psychometric evaluation. Br J Ophthalmol.

[8] Nutheti R, Shamanna BR, Nirmalan PK, Keeffe JE, Krishnaiah S, Rao GN (2006). Impact of impaired vision and eye disease on quality of life in Andhra Pradesh. Invest Ophthalmol Vis Sci.

[9] Fletcher A, Vijaykumar V, Selvaraj S, Thulasiraj RD, Ellwein LB (1998). The Madurai intraocular lens study. III: visual functioning and quality of life outcomes. Am J Ophthalmol.

[10] Finger RP, Kupitz DG, Fenwick E, Balasubramaniam B, Ramani RV, Holz FG (2012). The impact of successful cataract surgery on quality of life, household income and social status in South India. PLoS One.

[11] Khan A, Amitava AK, Rizvi SAR, Siddiqui Z, Kumari N, Grover S (2015). Cost-effectiveness analysis should continually assess competing health care options especially in high volume environments like cataract surgery. Indian J Ophthalmol.

[12] Murthy GVS, Gupta SK, Thulasiraj RD, Viswanath K, Donoghue EM, Fletcher AE (2005). The development of the Indian vision function questionnaire: questionnaire content. Br J Ophthalmol.

[13] Finger RP, Kupitz DG, Holz FG, Balasubramaniam B, Ramani RV, Lamoureux EL (2011). The impact of the severity of vision loss on vision-related quality of life in India: an evaluation of the IND-VFQ-33. Invest Ophthalmol Vis Sci.

[14] Cohen J (1988). Statistical power analysis for the behavioral sciences. 2nd edition. Abingdon-on-Thames, U.K.: Routledge.

[15] Linacre JM (1994). Sample size and item calibration stability. Rasch Meas Trans..

[16] Holladay JT (2004). Visual acuity measurements. J Cataract Refract Surg.

[17] Massof RW (2011). Understanding Rasch and item response theory models: applications to the estimation and validation of interval latent trait measures from responses to rating scale questionnaires. Ophthalmic Epidemiol.

[18] Cappelleri JC, Jason Lundy J, Hays RD (2014). Overview of classical test theory and item response theory for the quantitative assessment of items in developing patient-reported outcomes measures. Clin Ther.

[19] Petrillo J, Cano SJ, McLeod LD, Coon CD (2015). Using classical test theory, item response theory, and Rasch measurement theory to evaluate patient-reported outcome measures: a comparison of worked examples. Value Health J Int Soc Pharmacoeconomics Outcomes Res.

[20] Wright B, Linacre J (1994). Reasonable mean-square fit values. Rasch Meas Trans.

[21] Wright BD, Stone M. Measurement essentials. Wilmington: Wide Range Inc.; 1999.

[22] Thompson B (2004). Exploratory and confirmatory factor analysis: understanding concepts and applications. American Psychological Association.

[23] Masters GN (1982). A Rasch model for partial credit scoring. Psychometrika.

[24] Snyder CF, Aaronson NK, Choucair AK, Elliott TE, Greenhalgh J, Halyard MY (2012). Implementing patient-reported outcomes assessment in clinical practice: a review of the options and considerations. Qual Life Res Int J Qual Life Asp Treat Care Rehabil.

[25] Kroenke K, Monahan PO, Kean J (2015). Pragmatic characteristics of patient-reported outcome measures are important for use in clinical practice. J Clin Epidemiol.

[26] Pesudovs K, Garamendi E, Keeves JP, Elliott DB (2003). The activities of daily vision scale for cataract surgery outcomes: re-evaluating validity with Rasch analysis. Invest Ophthalmol Vis Sci.

[27] Skiadaresi E, Ravalico G, Polizzi S, Lundström M, González-Andrades M, McAlinden C. The Italian Catquest-9SF cataract questionnaire: translation, validation and application. Eye Vis. 2016;3. doi: 10.1186/s40662-016-0043-9. 10.1186/s40662-016-0043-9 PMC4848863 27127797

[28] McAlinden C, Gothwal VK, Khadka J, Wright TA, Lamoureux EL, Pesudovs K (2011). A head-to-head comparison of 16 cataract surgery outcome questionnaires. Ophthalmology.

