# Supplementary figures and images for: Development of a rapid point-of-care patient reported outcome measure for cataract surgery in India
Source: Health Qual Life Outcomes. 2018 Jan 30;16:25. doi: 10.1186/s12955-018-0855-5 (PMC5789621; doi:10.1186/s12955-018-0855-5)

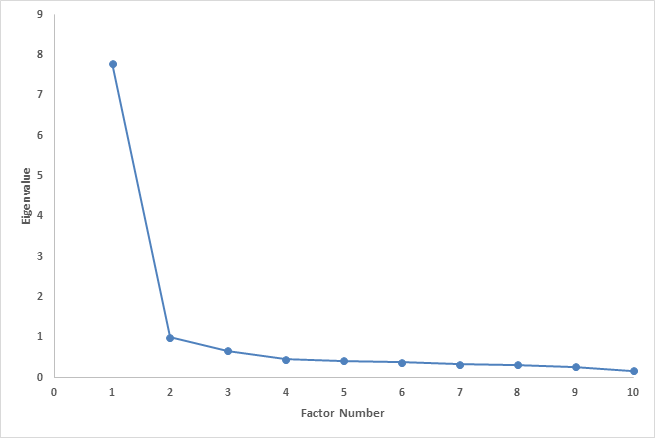

Supplement: Supplementary file 1 — Scree Plot of Exploratory Factor Analysis. This plot illustrates that the first eigenvalue of the exploratory factor analysis was the only value that was greater than 1. This suggests a unidimensional model. (TIFF 929 kb) [file 12955_2018_855_MOESM1_ESM.tif]
