# Supplementary material for: Development of a rapid point-of-care patient reported outcome measure for cataract surgery in India
Source: Health Qual Life Outcomes. 2018 Jan 30;16:25. doi: 10.1186/s12955-018-0855-5 (PMC5789621; doi:10.1186/s12955-018-0855-5)
Supplement: Supplementary file 2 — Supplementary tables from Rasch Analysis. These tables report for each of the 12 survey items: the average visual functioning estimates for each response category; category thresholds; and misfit statistics. (DOCX 19 kb) [file 12955_2018_855_MOESM2_ESM.docx]

**Additional File 2: Supplementary tables from Rasch Analysis**

These tables report for each of the 12 survey items: the average visual functioning estimates for each response category; category thresholds; and misfit statistics.

| **Observed Mean Category Estimates (theta)** | | | | |
| --- | --- | --- | --- | --- |
|  | **Observed Category Mean Estimate** | | | |
| **Item** | **Category 1** | **Category 2** | **Category 3** | **Category 4** |
| 1. Problem climbing stairs | - | 0.20 | 0.82 | 2.89 |
| 2. Problem making out bumps | - | -0.10 | 1.00 | 2.99 |
| 3. Problem seeing animals or vehicles | - | -0.45 | 0.77 | 2.87 |
| 4. Problem recognizing faces | - | -0.03 | 0.68 | 2.70 |
| 5. Problem seeing outside in bright light | - | 0.42 | 1.54 | 3.28 |
| 6. Frightened to go out | 0.82 | -0.39 | 0.90 | 2.75 |
| 7. Enjoy social functions less | - | 0.07 | 0.50 | 2.66 |
| 8. Ashamed can't see | -0.65 | 0.01 | 1.09 | 2.78 |
| 9. Dazzled in bright light | - | 0.42 | 2.03 | 3.04 |
| 10. Vision blurred in sunlight | -1.01 | 0.07 | 1.43 | 3.29 |
| 11. Bright light hurt eyes* | - | -0.12 | 1.95 | 2.46 |
| 12. Blurred vision | -0.11 | 0.48 | 1.95 | 3.74 |

*item removed from final instrument

| **Partial Credit Model Category Threshold Estimates** | | | |
| --- | --- | --- | --- |
|  | **Partial Credit Model Threshold Estimates** | | |
| **Item** | **Threshold 1** | **Threshold 2** | **Threshold 3** |
| 1. Problem climbing stairs | -1.68 | 0.70 | - |
| 2. Problem making out bumps | -1.06 | 1.34 | - |
| 3. Problem seeing animals or vehicles | -1.17 | 0.87 | - |
| 4. Problem recognizing faces | -0.89 | 0.35 | - |
| 5. Problem seeing outside in bright light | 0.14 | 2.26 | - |
| 6. Frightened to go out | -2.40 | -1.30 | 0.70 |
| 7. Enjoy social functions less | -0.87 | 0.13 | - |
| 8. Ashamed can't see | -2.03 | -1.19 | 0.97 |
| 9. Dazzled in bright light | -0.41 | 3.17 | - |
| 10. Vision blurred in sunlight | -2.33 | -0.27 | 2.05 |
| 11. Bright light hurt eyes* | -1.22 | 1.28 | - |
| 12. Blurred vision | -2.33 | 0.22 | 3.46 |

*item removed from final instrument

| **Item Category Misfit Statistics** | | | | | | | |  |
| --- | --- | --- | --- | --- | --- | --- | --- | --- |
|  | **Category 1** | | **Category 2** | | **Category 3** | | **Category 4** | |
|  | **Infit MNSQ** | **Outfit MNSQ** | **Infit MNSQ** | **Outfit MNSQ** | **Infit MNSQ** | **Outfit MNSQ** | **Infit MNSQ** | **Outfit MNSQ** |
| **Item** |  |  |  |  |  |  |  |  |
| 1 | - | - | 1.10 | 1.11 | 0.83 | 0.46 | 0.87 | 0.90 |
| 2 | - | - | 0.95 | 0.90 | 0.82 | 0.64 | 0.84 | 0.87 |
| 3 | - | - | 0.75 | 0.62 | 0.76 | 0.42 | 0.79 | 0.83 |
| 4 | - | - | 1.10 | 1.12 | 0.82 | 0.45 | 0.96 | 0.98 |
| 5 | - | - | 1.12 | 1.24 | 0.84 | 0.66 | 1.01 | 1.00 |
| 6 | 2.00 | 8.25 | 0.81 | 0.71 | 0.91 | 0.76 | 0.97 | 0.98 |
| 7 | - | - | 1.20 | 1.81 | 0.75 | 0.45 | 0.83 | 0.88 |
| 8 | 1.09 | 0.99 | 1.05 | 1.30 | 0.99 | 0.88 | 1.14 | 1.07 |
| 9 | - | - | 1.03 | 1.03 | 1.28 | 1.33 | 1.51 | 1.51 |
| 10 | 0.81 | 0.80 | 0.72 | 0.64 | 0.72 | 0.59 | 0.81 | 0.85 |
| 11* | - | - | 0.94 | 0.86 | 1.79 | 3.21 | 1.72 | 1.59 |
| 12 | 1.28 | 1.39 | 0.88 | 0.87 | 0.87 | 0.84 | 0.75 | 0.77 |

*item removed from final instrument
